# Novel hybrid tri-polymer hyalurosomes: unlocking next-generation trans-tympanic therapeutics through multi-Scale evaluations

**DOI:** 10.1016/j.ijpx.2025.100425

**Published:** 2025-10-26

**Authors:** Sadek Ahmed, Heba Attia, Osama Saher

**Affiliations:** a Department of Pharmaceutics and Industrial Pharmacy, Faculty of Pharmacy, Cairo University, Cairo, Egypt; b Department of Microbiology and Immunology, Faculty of Pharmacy, Cairo University, Cairo, Egypt; c Department of Laboratory Medicine, Karolinska Institute, Stockholm, Sweden; Department of Cellular Therapy and Allogeneic Stem Cell Transplantation (CAST), Karolinska University Hospital Huddinge and Karolinska Comprehensive Cancer Center, Stockholm, Sweden

**Keywords:** Ciprofloxacin, Hybrid tri-polymer hyalurosomes, Mucoadhesion, Biofilm inhibition, minimum bactericidal concentration, Trans-tympanic permeation

## Abstract

Acute otitis media (AOM) remains one of the most common middle ear infections, particularly in children, necessitating advanced non-invasive therapeutic strategies. This study introduces Novel Hybrid Tri-Polymer Hyalurosomes, an innovative vesicular system designed to enhance trans-tympanic delivery of ciprofloxacin (CFX). The nanosystems were fabricated using the ethanol injection technique and optimized via a 2^3^ factorial design, evaluating the effects of hyaluronic acid (HA): drug ratio (Factor-A), surfactant: HA ratio (Factor-B), and L121: Brij L4 ratio (Factor-C) on critical quality attributes. The optimized formula, selected using a high desirability index (0.996), exhibited high entrapment efficiency (EE%) of 90.28 %, small particle size (PS) of 218.15 nm, and promising zeta potential (ZP) of −40.4 mV. Transmission electron microscopy (TEM) confirmed the uniform spherical morphology of the optimized formula, which also exhibited a characteristic bi-phasic sustained release profile and pseudoplastic rheological behavior, enhancing ease of application and retention at the administration site. Moreover, the formulation demonstrated excellent storage stability and markedly improved mucoadhesive properties. Ex vivo permeation studies revealed a 2.53-fold enhancement ratio compared to CFX solution. Microbiological assessments showed significantly lower minimum inhibitory concentration (MIC) and minimum bactericidal concentration (MBC) values against *Pseudomonas aeruginosa* and *Staphylococcus aureus*, along with superior biofilm inhibition activity. Confocal laser scanning microscopy (CLSM) confirmed deeper tissue penetration, consistent with the permeation findings. Additionally, in vivo histopathological evaluation demonstrated the safety of the optimized formula with no observable tissue irritation or damage. Collectively, Novel Hybrid Tri-Polymer Hyalurosomes present a promising non-invasive trans-tympanic delivery platform, holding significant potential for advancing AOM therapy.

## Graphical abstract

**Figure:**
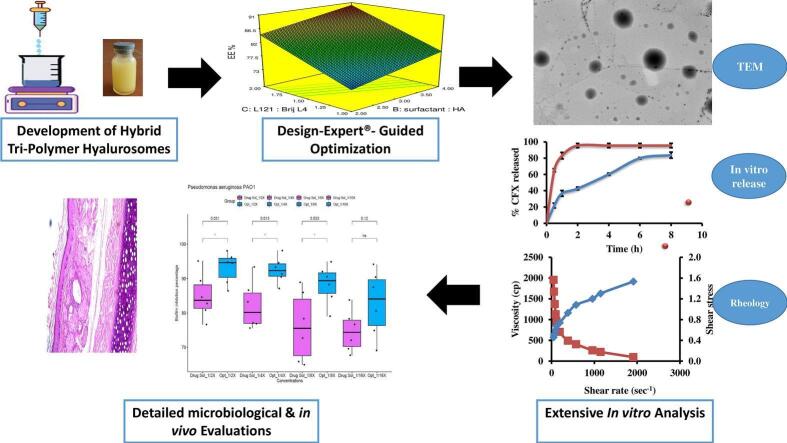

## 1 Introduction

Acute otitis media (AOM) is among the most prevalent infections in children, characterized by inflammation of the middle ear and often triggered by bacterial pathogens following upper respiratory tract infections. The condition contributes to significant morbidity worldwide, with recurrent episodes in early childhood potentially leading to conductive hearing loss, delayed speech development, and, in severe cases, intracranial complications such as mastoiditis and meningitis (Al-Mahallawi et al., 2014; Zeng et al., 2014). The standard management of acute otitis media (AOM) relies largely on systemic administration of broad-spectrum oral antibiotics. While effective, this approach often results in extensive systemic exposure, which can lead to adverse effects and contribute to the emergence of antibiotic resistance (Khoo et al., 2013; Younes et al., 2024). In contrast, localized trans-tympanic delivery of antimicrobial agents represents a more advanced, non-invasive alternative, as it allows higher drug levels at the site of infection while minimizing systemic side effects (Yang et al., 2017; Ahmed et al., 2025e). However, the clinical application of topical therapy is limited when the tympanic membrane (TM) remains intact, since its outermost stratum corneum forms a highly resistant barrier that restricts drug permeation. This reduced permeability not only lowers drug bioavailability but also compromises the therapeutic concentration achieved within the middle ear cavity (Al-Mahallawi et al., 2017). To address these challenges, the development of formulations capable of traversing an intact TM is crucial. The swift advancements in nanotechnology have revolutionized the field of drug delivery, introducing cutting-edge strategies designed to overcome the inherent limitations of traditional pharmaceutical formulations (Bazaz et al., 2021; Elmahboub et al., 2025). They have enabled the design of vesicular systems capable of enhancing trans-tympanic drug delivery by modulating particle size, surface characteristics, and deformability (Al-Mahallawi et al., 2017). In recent years, a variety of nanocarrier system, such as proniosomes (Younes et al., 2024), glycerosomes (Magdy et al., 2022), and glycerylated vesicles (Adwan et al., 2015), have been investigated to improve TM permeability and boost local drug bioavailability, offering promising prospects for non-invasive AOM therapy.

In this context, we introduce a novel Hybrid Tri-Polymer Hyalurosomal system tailored to enhance non-invasive delivery of ciprofloxacin (CFX) across the intact TM. This vesicular platform leverages the synergistic functionality of three distinct components. Pluronic® L121, a block copolymer consisting of poly(ethylene oxide)-poly(propylene oxide)-poly(ethylene oxide) (PEO–PPO–PEO), known for its amphiphilic properties. It facilitates permeation across biological barriers, and contributes to the deformability of the vesicles (Ahmed et al., 2021). Brij L4, a non-ionic surfactant that imparts structural stability, improves the interfacial behavior of vesicles, and supports encapsulation efficiency. Brij L4 also aids in modulating surface charge to optimize cellular and mucosal interactions (Sarkar and De, 2022; Younes et al., 2024). Hyaluronic Acid (HA), a naturally occurring biopolymer with high mucoadhesive potential and water retention capability. It enhances residence time on the TM, supports vesicle hydration, and contributes to anti-inflammatory and tissue-regenerative effects at the infection site (Fahmy et al., 2021; Nemr et al., 2023). These three components collectively form a versatile nanocarrier system capable of delivering ciprofloxacin with enhanced encapsulation, prolonged retention, and sustained release for effective AOM management.

Ciprofloxacin, a broad-spectrum fluoroquinolone antibiotic, was selected for its efficacy against common AOM pathogens (e.g., *Pseudomonas aeruginosa, Staphylococcus aureus*), relatively low molecular weight (331.4 Da), and favorable lipophilicity (log P ≈ 0.28), making it highly compatible with vesicular encapsulation and trans-epithelial delivery (Al-Mahallawi et al., 2014; Ahmed et al., 2025e). Its antibacterial mechanism involves disrupting the function of bacterial DNA gyrase, a critical enzyme responsible for supercoiling and maintaining DNA topology, ultimately leading to destabilization of the bacterial genome and degradation through exonuclease-mediated cleavage (Campoli-Richards et al., 1988).

In this study, Hybrid Tri-Polymer Hyalurosomes were fabricated via the ethanol injection method and systematically optimized using a 2^3^ factorial design to investigate the effects of HA: drug ratio, surfactant: HA ratio, and L121: Brij L4 ratio on critical quality attributes. The optimized formulation was evaluated for physicochemical characteristics, morphology, rheological and mucoadhesive behavior, drug release kinetics, ex vivo permeation, microbiological efficacy, and in vivo safety. This work pioneers a novel vesicular platform that unites structural flexibility, enhanced tissue permeation, and prolonged TM adhesion, offering a promising non-invasive strategy for targeted AOM treatment and potentially transforming the clinical landscape of pediatric middle ear infections.

## 2 Material and methods

### 2.1 Materials

Ciprofloxacin was kindly donated by El Nasr Pharmaceutical Co. (Cairo, Egypt). Hyaluronic acid, Brij L4, Pluronic® L121, Rhodamine B, and dialysis membranes (molecular weight cut-off 14,000 Da) were obtained from Sigma-Aldrich (USA). Egg-derived lecithin (phosphatidylcholine, 72 % purity) was purchased from Fisher Scientific (Leicestershire, UK). El Nasr Pharmaceutical Chemicals Company (Cairo, Egypt) also supplied disodium hydrogen phosphate, potassium dihydrogen phosphate, sodium chloride, and 95 % ethanol. Unless otherwise specified, all additional chemicals and solvents used in the present work were of analytical grade and utilized without any further purification.

### 2.2 Animal selection and housing conditions

Healthy adult male albino rabbits (average weight 2 ± 0.5 kg) were used in this study. The animals were housed individually in well-ventilated cages under controlled laboratory conditions (25 ± 2 °C) with an alternating 12-h light and dark cycle. They were maintained on a standard commercial diet and had unrestricted access to fresh drinking water. Prior to the start of the experiments, all animals were carefully examined by a veterinarian to confirm their general health status and to exclude any pre-existing otic infections or inflammatory conditions. All animal-handling and experimental procedures complied with the highest ethical standards and were approved by the Research Ethics Committee for Experimental and Clinical Studies, Faculty of Pharmacy, Cairo University, Egypt (Approval No. PI 3773). The study protocols were carried out in strict accordance with the Guide for the Care and Use of Laboratory Animals (US National Institutes of Health, NIH Publication No. 85–23, revised 2011). Furthermore, the work fully adhered to the ARRIVE guidelines, ensuring accurate reporting and the welfare of the animals involved.

### 2.3 Study design and factorial scheme

In the present investigation, CFX-loaded Novel Hybrid Tri-Polymer Hyalurosomes were systematically developed and optimized using a 2^3^ factorial experimental design. Three critical formulation variables were selected based on prior screening studies: the hyaluronic acid (HA): drug ratio (Factor A), the surfactant: HA ratio (Factor B), and the Pluronic L121: Brij L4 ratio (Factor C). Each factor was evaluated at two predetermined levels to enable robust analysis. To determine the optimal formulation, four key quality attributes were assessed as dependent responses: entrapment efficiency (EE%, Y₁), particle size (PS, Y₂), poly-dispersity index (PDI, Y₃), and zeta potential (ZP, Y₄). These parameters were carefully chosen to ensure the nanosystem possessed the desired physicochemical characteristics. The factorial design matrix, along with the specific factor levels and optimization constraints for each response, is presented in Table 1. All formulations generated from the design were prepared and evaluated, with their detailed compositions and recorded responses summarized in Table 2. Statistical modelling and response surface analyses were conducted using Design-Expert® software (Stat-Ease Inc., Minneapolis, MN, USA), which enabled precise assessment of the influence of each factor on the measured responses, thereby guiding the selection of the optimized formulation (Ahmed et al., 2025g; El Hassab et al., 2025).

**Table 1:** Factorial Design Framework: Key Parameters, Response Metrics, and Target Objectives.

Factor (independent variable)	Level	
	-1	+1
A: HA: Drug ratio	1.5:1	3:1
B: Surfactant: HA ratio	2:1	4:1
C: L121: Brij L4 ratio	1:1	2:1
**Response (dependent variable)**	**Desirability constraints**	
Y1: EE %	Maximize	
Y2: PS (nm)	Minimize	
Y3: PDI	Minimize	
Y4: ZP (absolute value) (mV)	Maximize	

**Abbreviations:** EE %, percent entrapment efficiency; HA, Hyaluronic acid; PDI, poly-dispersity index; PS, particle size; ZP, zeta potential.

**Table 2:** Formulation Profiles and Measured Responses of CFX-Loaded Hybrid Tri-Polymer Hyalurosomes (*n* = 3, mean ± SD).

Formula	Factors						
	A: HA: Drug ratio	B: Surfactant: HA ratio	C: L121: Brij L4 ratio	Y1: EE % (Mean ± SD)	Y2: PS (nm) (Mean ± SD)	Y3: PDI (Mean ± SD)	Y4: ZP (mV) (Mean ± SD)
F1	1.5	2	2	83.92 ± 0.78	151.95 ± 1.20	0.24 ± 0.01	−26.25 ± 2.62
F2	3	4	1	80.06 ± 1.60	212.55 ± 3.89	0.36 ± 0.04	−38.00 ± 0.57
F3	1.5	4	1	79.11 ± 0.58	179.60 ± 3.96	0.40 ± 0.05	−25.50 ± 4.81
F4	1.5	2	1	75.35 ± 4.13	161.60 ± 3.39	0.50 ± 0.04	−22.00 ± 0.85
F5	3	4	2	90.29 ± 1.12	218.15 ± 2.33	0.40 ± 0.08	−40.40 ± 3.82
F6	3	2	1	71.72 ± 1.02	173.40 ± 5.23	0.38 ± 0.03	−34.35 ± 4.60
F7	3	2	2	86.23 ± 3.64	174.60 ± 2.83	0.29 ± 0.03	−38.15 ± 0.92
F8	1.5	4	2	89.08 ± 2.19	191.85 ± 9.40	0.36 ± 0.01	−28.45 ± 1.20

**Abbreviations:** CFX, Ciprofloxacin; EE %, percent entrapment efficiency; HA, Hyaluronic acid; PDI, poly-dispersity index; PS, particle size; ZP, zeta potential.

### 2.4 Development of hybrid tri-polymer hyalurosomes

Ciprofloxacin (CFX)-loaded Novel Hybrid Tri-Polymer Hyalurosomes were prepared using a modified ethanol injection technique (Abdelbari et al., 2024; Ahmed et al., 2025c), a reliable and efficient method for producing nanoscale vesicular systems. The organic phase was prepared by dissolving 10 mg of CFX, 50 mg of phosphatidylcholine (PC), and the required amount of Brij L4 and/or Pluronic L121 (according to the factorial design) in 10 mL of absolute ethanol. Each formulation incorporated the specified polymeric ratios: HA: drug ratio at either 1.5:1 or 3:1, surfactant: HA ratio at 2:1 or 4:1, and Pluronic L121: Brij L4 ratio at 1:1 or 2:1, as dictated by the design matrix. The mixture was maintained at 60 °C in a thermostatically controlled water bath (Crest Ultrasonics Corp., Trenton, USA) to ensure complete solubilization of the lipids and surfactants. In parallel, the aqueous phase (double distilled water) was preheated to the same temperature. Under continuous magnetic stirring, the ethanoic solution was slowly injected into twice its volume of the aqueous phase, triggering the self-assembly of uniform nanovesicles. Following vesicle formation, hyaluronic acid (HA) was gradually dispersed into the suspension by sprinkling, ensuring efficient hydration and homogeneous distribution within the vesicular network (Fahmy et al., 2021). Stirring was continued until a clear and stable dispersion was obtained. The formulations were allowed to equilibrate at room temperature before being stored at 4 °C for further characterization (Mosallam et al., 2021b; Albash et al., 2022). Complete removal of the residual ethanol was confirmed by the absence of any detectable odor (Ahmed et al., 2025f).

### 2.5 In vitro profiling of CFX-loaded hybrid tri-polymer hyalurosomes

#### 2.5.1 Encapsulation efficiency analysis

The encapsulation efficiency (EE%) of ciprofloxacin in the developed Hybrid Tri-Polymer Hyalurosomes was determined indirectly by quantifying the concentration of free, unincorporated drug present in the external aqueous phase. Briefly, freshly prepared dispersions were subjected to ultracentrifugation at 20,000 rpm for 60 min using an ultra-centrifuge (Sigma 3–30 KS, Sigma Laborzentrifugen GmbH, Germany) maintained at 4 °C to ensure effective sedimentation of the vesicles (Ahmed et al., 2025a). The resulting clear supernatant, representing the non-entrapped fraction of the drug, was carefully collected and analyzed using UV–visible spectrophotometry (Shimadzu UV-1601 PC, Kyoto, Japan) at the characteristic absorbance wavelength of 279 nm (Al-Mahallawi et al., 2014). Quantitative analysis was performed with reference to a previously validated calibration curve (R^2^ = 0.9991, *n* = 3). The encapsulation efficiency was subsequently calculated by subtracting the amount of free drug from the total drug content originally used in the formulation, following the equation below (Sayed et al., 2020; Ahmed et al., 2025a):

(1) $$\mathrm{EE}\%=\frac{\left(\mathrm{Total amount of}\;\mathrm{QER}-\mathrm{Unentrapped}\;\mathrm{QER}\right)}{\mathrm{Total amount of}\;\mathrm{QER}}\;x\;100$$

#### 2.5.2 Assessment of particle size and surface potential

The particle size (PS), poly-dispersity index (PDI), and zeta potential (ZP) of the developed ciprofloxacin-loaded Hybrid Tri-Polymer Hyalurosomes were evaluated to characterize their size distribution and surface charge properties (Teama et al., 2025). For analysis, a 50 μL aliquot of each freshly prepared formulation was precisely withdrawn and diluted with deionized water at a 1:100 ratio to provide optimal scattering conditions. The diluted samples were vortexed gently to ensure homogenous dispersion of vesicles and to prevent possible aggregation prior to measurement. The measurements were carried out using a Zetasizer instrument (Malvern Instruments, Worcestershire, UK) operated at 25 °C at a fixed angle of 173° (El Hosary et al., 2024; Fahmy et al., 2025).

### 2.6 Design-expert®-guided formulation optimization

The experimental design and optimization of the ciprofloxacin-loaded Hybrid Tri-Polymer Hyalurosomes were conducted using Design-Expert® software (Stat-Ease Inc., Minneapolis, MN, USA). The primary objective was to develop a nanosystem with a vesicle size (PS) maintained within the desired range, a low poly-dispersity index (PDI), and the highest possible entrapment efficiency (EE%). Additionally, maximizing the absolute value of the zeta potential (ZP) was prioritized to ensure enhanced colloidal stability and reduce the risk of vesicle aggregation. To achieve this, the effects of each independent formulation variable were statistically evaluated using analysis of variance (ANOVA) (Hussein et al., 2024). A numerical optimization strategy based on the desirability function was then applied to integrate all measured responses into a single composite desirability index, which ranges from 0 (least desirable) to 1 (most desirable). This approach allowed simultaneous assessment of all critical quality attributes, enabling the selection of the most robust formulation (Ibrahim et al., 2024). To ensure the accuracy and reliability of the optimization process, the experimentally observed values of the optimized formulation were compared with the values predicted by the statistical model, and the percentage deviation was calculated. A minimal deviation between the predicted and observed responses confirmed the robustness and reliability of the optimization methodology. The percentage deviation was computed using the following equation (Ahmed et al., 2022b; Ahmed et al., 2024b):

% Deviation = (**|**Predicted value − Observed value**|/**(Observed value)) × 100 (Eq.2).

This optimized nano-dispersion was used as such in all in vitro*,* ex vivo*, and* in vivo studies without any further modifications.

### 2.7 Extensive analysis of the optimized formulation

#### 2.7.1 Morphological analysis using TEM

The surface architecture of the optimized ciprofloxacin-loaded Hybrid Tri-Polymer Hyalurosomes were examined using Transmission Electron Microscopy (TEM, model JEM-1230; JEOL, Tokyo, Japan) operated at an accelerating voltage of 80 kV (Albash et al., 2021; Elgendy et al., 2024). Prior to imaging, the formulation was appropriately diluted with deionized water to achieve adequate particle dispersion. A small aliquot of the diluted sample was carefully deposited onto a carbon-coated copper grid and allowed to air-dry at room temperature. To enhance image contrast and visualize the vesicular morphology clearly, the grid was subjected to negative staining with 2 % (*w*/*v*) phosphotungstic acid. After complete drying, the prepared samples were directly visualized under the TEM (Elmahboub et al., 2024; Abdelhakeem et al., 2025).

#### 2.7.2 FTIR spectral analysis

Fourier Transform Infrared Spectroscopy (FTIR) was employed to investigate potential physicochemical interactions between ciprofloxacin (CFX) and the excipients, as well as to confirm its successful encapsulation within the optimized Hybrid Tri-Polymer Hyalurosome formulation (Elgendy et al., 2023; Ahmed et al., 2024a). The FTIR spectra of pure CFX, phosphatidylcholine (PC), hyaluronic acid (HA), and the optimized formulation were recorded using a Bruker FTIR spectrometer (Model 22, Coventry, UK). Prior to analysis, each sample was thoroughly dried and gently compressed into potassium bromide (KBr) discs. The spectra were collected at 25 °C over a scanning range of 4000–500 cm^−1^. Characteristic functional group peaks of the individual components were compared with those of the optimized formulation to detect any shifts in peak positions or changes in peak intensity (Younes et al., 2024).

#### 2.7.3 Assessment of mucoadhesive potential

The mucoadhesive properties of the optimized Hybrid Tri-Polymer Hyalurosome formulation were assessed using a 1 % w/v mucin solution. Equal volumes of the optimized formulation and the mucin dispersion (1:1 *v*/v) were mixed thoroughly and subjected to magnetic stirring for 5 min to ensure homogeneity (Tawfik et al., 2025). The resulting mixture was then allowed to equilibrate at ambient temperature overnight to enable interaction between the vesicles and the mucin (Nemr et al., 2024). The zeta potential (ZP) of the mucin solution and the optimized formulation were determined individually and compared to the ZP of the mucin–formulation mixture using a Zetasizer. A marked change in the ZP of the mixture relative to the individual components was interpreted as evidence of strong electrostatic interactions, reflecting the mucoadhesive capability of the optimized formulation (Albash et al., 2021; Ahmed et al., 2025f).

#### 2.7.4 In vitro release profile and kinetic modelling

The in vitro release behavior of the optimized ciprofloxacin-loaded Hybrid Tri-Polymer Hyalurosome formulation was evaluated and compared to that of a plain CFX solution using the dialysis bag method (Weng et al., 2020). Prior to the experiment, the dialysis membrane (molecular weight cut-off: 12,000–14,000 Da) was soaked overnight in phosphate-buffered saline (PBS, pH 7.4), which served as the release medium. A pre-measured amount of the optimized formulation or CFX solution, equivalent to 1 mg of CFX, was carefully enclosed within the hydrated dialysis membrane.

The sealed dialysis bags were immersed in amber glass bottles containing 50 mL of PBS (pH 7.4) and maintained at 37 ± 0.5 °C in a thermostatically controlled shaking water bath operating at 100 rpm to mimic physiological conditions. At predetermined intervals (0.5, 1, 2, 4, 6, and 8 h), 3 mL aliquots were withdrawn from the release medium and immediately replaced with an equal volume of fresh pre-warmed PBS to maintain sink conditions (Ahmed and Badr-Eldin, 2019). The cumulative percentage of CFX released was determined using UV–visible spectrophotometry at λ_max_ 279 nm (*n* = 3, R^2^ = 0.9991). The obtained release profiles were analyzed using various kinetic models, including zero-order, first-order, Higuchi's diffusion model, and Korsmeyer–Peppas equations, to elucidate the drug release mechanism. The model providing the highest regression coefficient (R^2^) was considered the best fit for describing the release kinetics of the optimized formulation (Abdelhakeem et al., 2025; Ahmed et al., 2025b).

#### 2.7.5 Viscosity and flow characterization

The rheological behavior of the optimized formulation was comprehensively assessed using a rotational cone and plate viscometer (Brookfield DV3THB, spindle CPE-41) operated at a controlled temperature of 25 ± 1 °C. Precisely 0.5 g of the sample was placed on the plate, and rotational speeds were sequentially increased from 0.5 to 100 rpm, pausing for 10 s between each speed increment to ensure equilibrium (Younes et al., 2025). Only data with torque values within the 10 %–100 % range were considered valid for analysis. The collected data were utilized to construct a flow curve representing the interrelationship between shear rate, shear stress, and viscosity, offering insight into the formulation's viscoelastic properties. To interpret its flow characteristics, the Power Law model was applied, defined by the equation (Nemr et al., 2021; Ahmed et al., 2025g):

Ʈ = KƔ^n^ (Eq.3).

where Ʈ denotes shear stress, Ɣ is shear rate, K is the consistency index, and n is the flow behavior index. This model allows classification of fluid behavior where *n* = 1 corresponds to Newtonian flow, *n* < 1 indicates shear-thinning (pseudoplastic) behavior, and *n* > 1 reflects shear-thickening (dilatant) behavior. Further rheological characterization involved fitting the experimental data to advanced non-Newtonian models, including Bingham, Casson, and Carreau, with model accuracy determined by comparing their correlation coefficients (R^2^ values). These analyses not only confirmed the non-Newtonian nature of the formulation but also elucidated its structural response under varying shear conditions, which is crucial for predicting its performance during administration and handling Hegazy et al., 2025.

#### 2.7.6 Impact of storage

To investigate the short-term stability of the optimized hybrid tri-polymer hyalurosomal formulation encapsulating ciprofloxacin (CFX), samples were stored in tightly sealed glass vials under refrigerated conditions (5 ± 3 °C) for a duration of three months. This evaluation aimed to determine the formulation's ability to retain its physicochemical integrity and therapeutic functionality over time (Sayed et al., 2021a). Following the storage period, the formulation was re-analyzed for key quality attributes, including particle size (PS), zeta potential (ZP), and entrapment efficiency (EE%) (Ahmed et al., 2025b). These parameters were compared to their initial values using one-way analysis of variance (ANOVA) to detect any statistically significant deviations. In addition, visual inspection was conducted to confirm the absence of aggregation, phase separation, or precipitation—indicative of physical stability (Al-Mahallawi et al., 2017). The in vitro drug release profile was also reassessed under the same conditions employed in the original study. To quantify the similarity between pre- and post-storage release patterns, the similarity factor (f₂) was calculated according to the following equation (Adel et al., 2021):

(4) $$f_{2}=50.\log\{{\left[1+\left(\frac{1}{n}\right)\sum_{t=1}^{n}{\left(R_{t}-T_{t}\right)}^{2}\right]}^{-0.5}.100$$

where Rₜ and Tₜ denote the cumulative percentage of CFX released at each sampling time point before and after storage, respectively. An f₂ value greater than 50 was interpreted as evidence of no significant change in release behavior, affirming the formulation's release consistency (Abdelbary et al., 2016).

### 2.8 Microbiological efficacy assessment

#### 2.8.1 Selection of the bacterial strains and the antibiotic compound

*Staphylococcus aureus* and *Pseudomonas aeruginosa* were selected as representative bacterial strains due to their well-documented clinical relevance in recurrent and chronic otitis media, particularly in cases associated with tympanic membrane perforation or tympanostomy tubes (Sonsuwan et al., 2016; Rath et al., 2017; Jo et al., 2022). These pathogens are frequently implicated in chronic suppurative otitis media (CSOM) and are known for their multidrug resistance and biofilm-forming capabilities, which significantly complicate treatment outcomes (Abdulhaq et al., 2020; Assefa and Amare, 2022). Ciprofloxacin, a fluoroquinolone antibiotic, was chosen as the therapeutic agent in this study because of its potent activity against *P. aeruginosa* and *S. aureus*, including biofilm-forming strains. Its broad-spectrum efficacy and established clinical use in otic infections make it a suitable model compound to evaluate the performance and therapeutic potential of the developed formulation (Chalkley and Koornhof, 1985; Bartzatt et al., 2010; Nwabuife et al., 2022; Osman et al., 2023).

#### 2.8.2 Evaluating the antibacterial efficacy through MIC and MBC determination

The antibacterial activity of the optimized formulation was assessed by determining its minimum inhibitory concentration (MIC) and minimum bactericidal concentration (MBC) following the broth microdilution method as recommended by the Clinical and Laboratory Standards Institute (CLSI) guidelines (Humphries et al., 2018). In sterile 96-well microtiter plates with U-shaped wells, two-fold serial dilutions of both the ciprofloxacin solution and the optimized Hybrid Tri-Polymer Hyalurosome formulation were prepared over a concentration range of 250–0.122 μg/mL, using 75 μL of double-strength Mueller-Hinton Broth (MHB). Subsequently, 15 μL of standardized bacterial suspensions (*Pseudomonas aeruginosa* PAO1 or *Staphylococcus aureus* USA300; inoculum size 10^5^–10^6^ CFU/mL) was added to each well. Appropriate controls were included: a positive control for bacterial growth, a negative control for media sterility, and a blank control to exclude any antibacterial effect of the excipients. After a 24 h incubation at 37 °C, the wells were visually examined, and the absorbance was measured at 600 nm using a microtiter plate reader (Biotek, Synergy 2, USA). The MIC was recorded as the lowest concentration with no visible bacterial growth. All experiments were performed in biological and technical triplicates.

For MBC determination, the same broth microdilution protocol was followed (Humphries et al., 2018). After the 24  h incubation, 10 μL aliquots from wells, above the MIC concentration, was spotted onto Mueller-Hinton Agar (MHA) plates and incubated at 37 °C for an additional 24 h. The MBC was defined as the lowest concentration showing complete absence of bacterial colony formation. The experiments were carried out in triplicate to ensure reproducibility.

#### 2.8.3 Assessment of the biofilm inhibition

The anti-biofilm activity of both the ciprofloxacin solution and the optimized Hybrid Tri-Polymer Hyalurosome was assessed against the tested bacterial strains using a microtiter plate assay (O'toole, 2011; Haney et al., 2021). Briefly, 50 μL of standardized bacterial suspension (10^8^ CFU/mL in Mueller-Hinton Broth, (MHB) for *Pseudomonas aeruginosa* PAO1 or in Tryptic soy broth (TSB) for *Staphylococcus aureus* USA300) was mixed with 50 μL of either the drug solution or the optimized formulation in sterile, non-pyrogenic, flat-bottom 96-well polystyrene microtiter plates. The final concentrations tested corresponded to ^1^/_16_, ^1^/_8_, ^1^/_4_, and ^1^/_2_ X, where X represents the previously determined MIC value. Appropriate positive and negative controls were included in each experiment.

The plates were incubated under static conditions at 37 °C for 24 h. Following incubation, the optical density (OD₆₀₀) of the planktonic bacterial culture was recorded using an automated microtiter plate reader (Biotek, Synergy 2, USA). Supernatants were gently aspirated, and each well was rinsed twice with sterile phosphate-buffered saline (PBS, pH 7.4) before allowing the plates to dry completely. The dried biofilms were then stained with 125 μL of 0.5 % crystal violet solution for 30 min at room temperature. Excess stain was removed by washing the plates three times with sterile distilled water, followed by air-drying.

For quantification of the crystal violet-stained biofilms, 150 μL of 95 % ethanol was added to each well to solubilize the retained crystal violet, and the plates were incubated, with shaking at 110 rpm, for 15 min. The optical density of the solubilized stain was measured at OD₅₇₀ and normalized to OD₆₀₀ readings of the planktonic bacterial cultures. All assays were performed in triplicate and repeated independently three times. The percentage of biofilm inhibition was calculated using the following equation:

(5) $$\text{Biofilm inhibition}\%=\frac{\mathrm{OD}\;\text{Control}-\mathrm{OD}\;\text{Test}}{\mathrm{OD}\;\text{Control}}\times 100$$

### 2.9 Integrated ex vivo and in vivo assessments in animals

#### 2.9.1 Ex vivo permeation

The rabbit tympanic membrane was selected as an ex vivo*/*in vivo model due to its anatomical and structural similarities to the human tympanic membrane, particularly in terms of trilaminar architecture, relative thickness, and functional barrier properties (Ahmed et al., 2025e). While interspecies differences in size and exact composition exist, the rabbit model has been widely employed in otic drug delivery research because its permeability and physiological response closely approximate those of the human tympanic membrane (Younes et al., 2024). These features make it a reliable and translationally relevant model for preliminary evaluation of drug permeation and safety prior to advancing to human studies (Al-Mahallawi et al., 2017; Abdelbary et al., 2019). In our study, Male albino rabbits (weighing 2 ± 0.5 kg) were anesthetized using intramuscular injections of 35 mg/kg ketamine and 5 mg/kg xylazine prior to decapitation (Emad Eldeeb et al., 2019; Ahmed et al., 2024a). The bullae containing the tympanic membranes (TMs) were carefully excised following the technique outlined by Khoo et al. (Khoo et al., 2013). Each TM was examined under a dissection microscope (10× magnification) to confirm the absence of any structural defects before further testing (Al-Mahallawi et al., 2014). The extracted bullae were suspended vertically by a thread in a beaker containing 25 mL of phosphate-buffered saline (PBS, pH 7.4) as the receptor medium, ensuring that the medial surface of the TM was in direct contact with the receptor compartment. A defined dose of either 1 mg ciprofloxacin (CFX) from the optimized formulation or an equivalent dose from a CFX solution (control) was instilled into the external auditory meatus (EAM) to fully cover the lateral TM surface. The receptor medium was continuously stirred at 100 rpm using a magnetic stirrer and maintained at 37 °C to mimic physiological conditions (Abdelbary et al., 2019). At predetermined time intervals (0.5, 1, 2, 4, 6, 8 and 10 h), 2 mL samples were withdrawn and immediately replaced with an equal volume of fresh receptor medium to maintain sink conditions. Each collected sample was mixed with an equal volume of methanol, sonicated for 3 min to precipitate proteins, and centrifuged at 4000 rpm for 15 min. The concentration of CFX in the obtained supernatants was determined using UV–Vis spectrophotometry at λ_max_ 279 nm as described earlier. The cumulative amount of drug permeated per unit area (Q, μg/cm^2^) was calculated for each time interval. Furthermore, the flux (Jmax) and enhancement ratio (ER) were determined providing a comparative assessment of the optimized formulation's trans-tympanic delivery performance relative to the CFX solution using the following equations (Elmeshad and Mohsen, 2016; Ahmed et al., 2024b):

(6) $$J_{\max}=\frac{\mathrm{Amount of drug permeated}}{\mathrm{Time}\;X\;\mathrm{Area}}$$

(7) $$\mathrm{ER}=\frac{\text{Jmax}\;\mathrm{of optimum formula}}{\mathrm{Jmax of control}}$$

#### 2.9.2 Histological assessment of safety

To evaluate the local safety and tissue compatibility of the optimized Hybrid Tri-Polymer Hyalurosome formulation, a detailed histological analysis of the tympanic membrane was carried out (Younes et al., 2024). Three healthy male albino rabbits (average weight 2 ± 0.5 kg) were used for the study. Sterile normal saline was employed as a negative control for comparison. Each animal received 100 μL (approximately one drop) of either normal saline (instilled into the left ear) or the optimized formulation (instilled into the right ear) three times daily for one week (Abdelbary et al., 2019). At the end of the dosing period, the rabbits were anesthetized and humanely sacrificed as previously described. The tympanic membranes were carefully excised and immediately fixed in 10 % *v*/v formalin-saline solution until further processing. The tissues were then sequentially dehydrated in graded ethanol solutions, cleared with xylene, and embedded in molten paraffin wax at 56 °C for 24 h. Paraffin-embedded tissue blocks were sectioned using a precision microtome, and the obtained thin sections were mounted on clean glass slides. The sections were subsequently deparaffinized, rehydrated, and stained with hematoxylin and eosin (H&E) for microscopic evaluation. Histological examination was performed using a light microscope to assess any structural alterations, tissue irritation, or inflammatory response associated with the optimized formulation compared to the control (Bancroft et al., 2007; Abdelbary and Aboughaly, 2015).

#### 2.9.3 CLSM-based in vivo trans-tympanic uptake

Achieving deep trans-tympanic penetration is crucial for maximizing antibacterial efficacy; therefore, the permeation potential of the optimized Hybrid Tri-Polymer Hyalurosome formulation was evaluated using Rhodamine B (RhB) as a fluorescent tracer (Sayed et al., 2021a; Nemr et al., 2024). RhB was incorporated into the optimized formulation at a concentration of 0.1 % *w*/w, replacing ciprofloxacin. For comparison, an aqueous RhB solution (0.1 % *w*/w) served as the negative control. In the study, one ear of a healthy male albino rabbit (2 ± 0.5 kg) received a single drop (100 μL) of the RhB-loaded optimized formulation, while the contralateral ear was treated with the RhB aqueous solution. After 6 h, the animals were anesthetized and humanely sacrificed as described previously. The tympanic membranes (TMs) were carefully excised, rinsed to remove any residual formulation, and immediately preserved for imaging. The penetration depth of RhB into the TM tissue was visualized using confocal laser scanning microscopy (CLSM) (LSM 710; Carl Zeiss, Jena, Germany). Fluorescent imaging was performed using excitation at 485 nm (argon laser) and emission detection at 595 nm (helium‑neon laser). The resulting confocal images were processed and analyzed using LSM software version 4.2 (Carl Zeiss Microimaging, Jena, Germany) to determine and compare the extent of tissue permeation achieved by the optimized formulation versus the control (Younes et al., 2018; Ahmed et al., 2025b).

## 3 Results and discussion

### 3.1 Analysis of factorial design

To precisely optimize the formulation parameters influencing the physicochemical performance of the Hybrid Tri-Polymer Hyalurosomes, a 2^3^ factorial design was strategically implemented. This systematic design facilitated a comprehensive evaluation of three key formulation variables: the hyaluronic acid (HA): drug ratio, the surfactant: HA ratio, and the L121: Brij L4 ratio. Prior to finalizing the matrix, preliminary screening experiments were conducted to define the appropriate high and low bounds for each variable, ensuring practical feasibility. The factorial design enabled efficient exploration of the selected factors, with a minimal number of experimental trials (Zhang et al., 2010). Responses including entrapment efficiency (EE%), particle size (PS), poly-dispersity index (PDI), and zeta potential (ZP) were chosen as critical quality attributes to ensure optimal encapsulation, colloidal stability, and nanoscale dispersion that are essential for effective trans-tympanic delivery. The experimental data were statistically analyzed using Design-Expert® software, which facilitated model selection, prediction, and surface response visualization. The reliability of the generated models was confirmed through several statistical parameters: adequate precision values for all responses exceeded 4, indicating strong signal-to-noise ratios; coefficient of determination (R^2^) values were high, and the differences between predicted and adjusted R^2^ values remained below 0.2, confirming a robust and well-fitted model (Habib et al., 2018; Ahmed et al., 2025a). These metrics, detailed in Table 3, validate the model's predictive strength and reliability across the studied design space.

**Table 3:** Statistical Assessment of the Examined Responses.

Response	R^2^	Adjusted R^2^	Predicated R^2^	Adequate precision	Significant factors
EE %	0.9623	0.9341	0.8494	13.71	B,C
PS (nm)	0.9461	0.9057	0.7845	12.07	A,B
ZP (mV)	0.9969	0.9946	0.9876	51.04	A,B,C

**Abbreviations:** EE %, percent entrapment efficiency; PDI, poly dispersity index; PS, particle size; ZP, zeta potential.

#### 3.1.1 Statistical evaluation of EE%

Attaining high drug entrapment efficiency is essential for the success of nanocarrier-based otic formulations, especially for treating conditions like acute otitis media, where maximizing local drug retention at the site of infection is critical for therapeutic efficacy and reduced systemic exposure (Abdelbary et al., 2019). In this study, the entrapment efficiency (%EE) of the formulated Hybrid Tri-Polymer Hyalurosomes ranged from 71.72 ± 1.02 % to 90.29 ± 1.12 %, indicating excellent encapsulation capacity across the tested design space (Table 2). Statistical modelling using a 2^3^ factorial design revealed the following coded predictive equation:

EE%=81.97+0.10A+2.66B+5.41C

**Fig. 1:**
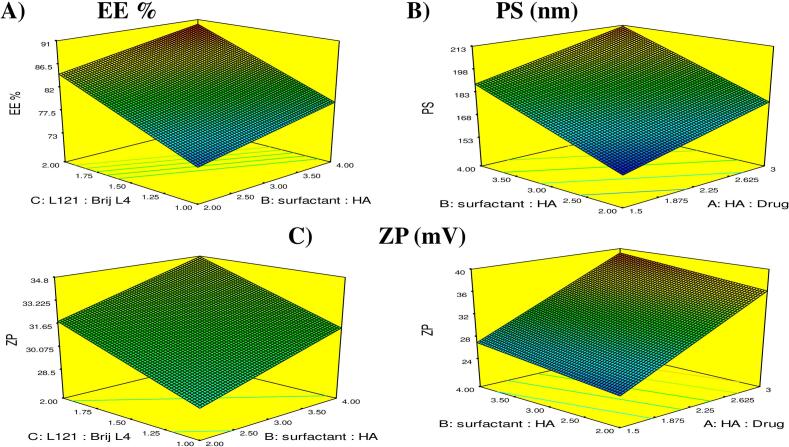
Response 3D plots for the effect of amount of HA: Drug ratio (Factor A), Surfactant: HA ratio (Factor B) and L121: Brij L4 ratio (Factor C) on (A) entrapment efficiency percentage (EE %), (B) particle size (PS) and (C) zeta potential (ZP).

Analysis of variance (ANOVA) confirmed that Factor B (surfactant: HA ratio) and Factor C (L121: Brij L4 ratio) had statistically significant positive effects on %EE (*p <* 0.05), whereas Factor A (HA: drug ratio) did not show a meaningful impact within the studied range (Fig. 1 **A**). The notable influence of Factor B suggests that increasing the proportion of surfactants relative to hyaluronic acid improves the encapsulation efficiency. This can be attributed to a more robust vesicular bilayer formed by enhanced surfactant interactions, which promotes tighter drug entrapment and prevents leakage. This balance is especially crucial given HA's hydrophilic nature, which, if overly dominant, may destabilize the lipid bilayer and compromise drug retention. Furthermore, high surfactant concentrations have the ability to enhance emulsifying capacity and stabilizing effect provided by the lipid matrix (Abdelbary and Fahmy, 2009; Ahmed et al., 2022a). More significantly, Factor C (L121: Brij L4 ratio) demonstrated the greatest enhancement effect on EE%, which can be rationalized by the distinct physicochemical properties of the surfactants involved. L121, having an HLB value of 1, is far more lipophilic than Brij L4 (HLB = 9), contributing to a more hydrophobic vesicle core that favours the retention of the molecules. Increasing the L121 proportion results in deeper entrapment within the bilayer structure and reduced diffusion into the aqueous phase. Furthermore, the steric bulk and low polarity of L121 may aid in stabilizing the vesicle matrix and limiting premature drug leakage (Abdelbary et al., 2017; Ahmed et al., 2025b). These findings highlight the crucial role of surfactant selection and ratio in modulating entrapment efficiency.

#### 3.1.2 Statistical evaluation of PS

The vesicle size of nanocarriers is a pivotal parameter that governs their biological performance, especially in non-invasive drug delivery systems such as trans-tympanic carriers. Smaller particles not only promote enhanced tissue penetration but also contribute to increased surface area, prolonged residence time, and better interaction with mucosal membranes (Safwat et al., 2017). In the current investigation, the particle size (PS) of the fabricated hybrid tri-polymer Hyalurosomes ranged between 151.95 ± 1.20 nm and 218.15 ± 2.33 nm (Table 2), placing them well within the ideal nanoscale range for mucosal drug delivery. Statistical evaluation revealed that Factor A (HA: Drug ratio) and Factor B (Surfactant: HA ratio significantly influenced the particle size (*p <* 0.05), while Factor C (L121: Brij L4 ratio) showed no substantial impact (*p >* 0.05). This is illustrated in **Fig. 1B** and reflected in the coded polynomial equation:

PS=182.96+11.71A+17.58B+1.17C

These findings indicate that increasing the proportion of either hyaluronic acid or surfactant leads to a noticeable enlargement in vesicle size. The enlargement effect associated with higher HA concentrations may stem from its intrinsic high molecular weight and strong anionic nature (Ahmed et al., 2025h). The abundant carboxylate groups of HA can form electrostatic and hydrogen bonding interactions with the phospholipid head groups, leading to the formation of a hydrated groups. This surface adsorption increases the hydrodynamic diameter of the vesicles, thereby elevating their overall particle size (Tran et al., 2014; Fahmy et al., 2021). Regarding Factor B, the rise in particle size with increased surfactant content can be attributed to its dual role in vesicle formation and stabilization. Higher surfactant levels typically lower the interfacial tension, facilitating the formation of more flexible and larger bilayers. This altered interfacial environment may lead to the formation of loosely packed bilayers, which can encapsulate more drug or aqueous phase, thus contributing to size expansion (Abdelbari et al., 2021). These observations collectively highlight the importance of carefully balancing the HA and surfactant ratios during formulation development.

#### 3.1.3 Statistical evaluation of PDI

The poly-dispersity index (PDI) is a fundamental parameter used to assess the uniformity of nanoparticle size distribution, which directly impacts the stability, reproducibility, and performance of nanoscale drug delivery systems. A lower PDI, approaching zero, indicates a highly homogeneous vesicle population, whereas values closer to one suggest broader size variability. In pharmaceutical nanocarriers, a PDI below 0.5 is generally accepted as indicative of a monodisperse system suitable for applications (Nemr et al., 2021; Nemr et al., 2022). In this study, the PDI values of the developed Hybrid Tri-Polymer Hyalurosomes were found to be within the range of 0.24 ± 0.01 to 0.50 ± 0.04 (Table 2), reflecting a consistently narrow distribution across all experimental trials. This indicates that the formulation process reliably produced vesicles of comparable sizes, which is advantageous for ensuring consistent drug release, bioavailability, and permeation across the tympanic membrane (Shalaby et al., 2022). Statistical evaluation via one-way ANOVA revealed that none of the formulation variables had a statistically significant effect on the PDI values (*p >* 0.05). Although the PDI parameter did not exhibit statistical sensitivity to the chosen formulation factors and was thus excluded from the final optimization criteria, its consistent values reinforce the reliability of the nanosystem.

#### 3.1.4 Statistical evaluation of ZP

Zeta potential (ZP) serves as a crucial indicator of the electrostatic stability of nano-vesicular systems. It reflects the surface charge on vesicles, which governs their colloidal behavior, including their propensity to repel each other and remain dispersed. Generally, nanocarriers with ZP values around ±30 mV exhibit sufficient repulsive forces to prevent aggregation, thereby promoting long-term stability (El Taweel et al., 2023). In the present investigation, the ZP of the formulations ranged between −22.00 ± 0.85 mV and − 40.40 ± 3.82 mV, as shown in Table 2, indicating satisfactory colloidal stability for mucosal application. A statistical assessment revealed that all three formulation variables exerted statistically significant positive effects on ZP (**Fig. 1C**). The relationship was described by the coded equation:

ZP=31.64+6.09A+1.45B+1.67C

The substantial influence of Factor A (HA: drug ratio) on the surface charge can be attributed to the anionic nature of hyaluronic acid. With increasing HA content, more carboxyl groups become available to interact electrostatically with the surface of the vesicles. This results in a thicker, negatively charged polymeric shell that increases the magnitude of ZP (in the negative direction), reinforcing the electrostatic barrier between particles and thus enhancing colloidal stability (Tran et al., 2014). Factor B, representing the ratio of surfactant to HA, also positively influenced ZP. As the surfactant content increased, the enhanced surface activity and structural arrangement likely promoted more effective coverage of the vesicle interface. This led to increased interfacial tension regulation and reduced vesicle coalescence (Ahmed et al., 2024b). As for Factor C, increasing the proportion of L121 relative to Brij L4 led to a significant increase in the absolute ZP value. Although L121 is more lipophilic (HLB = 1) compared to Brij L4 (HLB = 9), its bulkier molecular structure and longer hydrophobic chains introduce greater steric hindrance at the vesicle surface (Nour et al., 2016). This steric barrier plays a pivotal role in preventing close vesicle-vesicle interactions, reducing aggregation tendencies, and thus enhancing colloidal stability (Pradilla et al., 2015). Collectively, the findings confirm that all formulation components significantly contribute to the final surface charge of the system, and thus must be carefully balanced to optimize electrostatic stability (Daralnakhla et al., 2021).

### 3.2 Design-expert®-based optimization

The optimization of the Hybrid Tri-Polymer Hyalurosomes was accomplished through numerical analysis using the Design-Expert® software, which integrates a desirability function approach to statistically determine the most effective combination of formulation variables. This multi-criteria optimization tool evaluates each response based on its proximity to ideal targets, combining them into a single overall desirability score ranging from 0 (undesirable) to 1 (fully desirable) (Helal et al., 2024). Through this computational screening, the formulation that best satisfied the targeted physicochemical attributes (high entrapment efficiency, nanoscale particle size, low poly-dispersity index, and favorable zeta potential) was identified. The optimized formula was characterized by a HA to drug ratio of 3:1 (Factor A), a surfactant to HA ratio of 4:1 (Factor B), and a L121 to Brij L4 ratio of 2:1 (Factor C). This combination yielded an exceptionally high desirability value of 0.996, signifying a near-ideal balance between all critical quality attributes. The predictive model demonstrated excellent statistical strength, with minimal variation between the predicted and observed values for all measured responses, thereby affirming the robustness and reliability of the optimization process. These findings collectively validate the selection process for further development and evaluation (Yousry et al., 2020).

### 3.3 Extensive analysis of the optimized formulation

#### 3.3.1 Morphological analysis using TEM

**Fig. 2:**
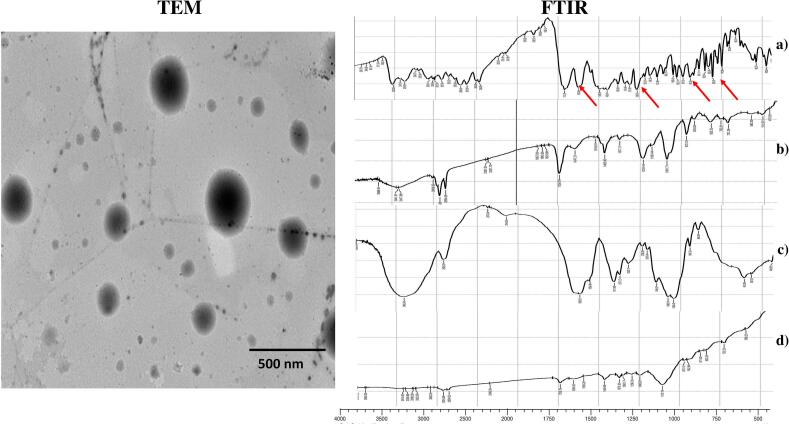
FTIR spectra of pure CFX, PC, HA and optimum formula. In addition to, TEM of the optimum formula, demonstrating uniformly spherical vesicles and the absence of characteristic ciprofloxacin peaks, which together support the physical encapsulation model.

The morphological examination of the optimized Hybrid Tri-Polymer Hyalurosomes using transmission electron microscopy (TEM) is depicted in Fig. 2. The micrograph reveals a population of uniformly spherical vesicles with narrow size distribution, reflecting the successful fabrication and nanoscale consistency of the system. The vesicles appear distinctly separated, with no visible signs of clumping, deformation, or fusion indicative of excellent colloidal stability and formulation integrity (Sayed et al., 2021b; Sakr et al., 2023). The well-dispersed morphology can be attributed to the synergistic stabilizing effect of the tri-polymeric matrix, which includes hyaluronic acid, L121 and Brij L4 blend. The presence of a negative surface charge, combined with steric hindrance conferred by the surfactants, likely contributed to repulsive inter-vesicular forces that prevented aggregation during and after formation (Nemr et al., 2022; Ahmed et al., 2025b). This morphological uniformity not only reflects the robustness of the formulation process but also reinforces the system's potential for consistent drug delivery across the tympanic membrane. Overall, the structural integrity and nanoscale architecture of the optimized Hyalurosomes support their suitability as a promising vehicle for non-invasive otic therapy.

#### 3.3.2 FTIR spectral analysis

FTIR spectroscopy was utilized to assess the chemical compatibility between ciprofloxacin (CFX) and the principal components of the Hybrid Tri-Polymer Hyalurosomes, while also serving as supportive evidence for the successful incorporation of the drug within the optimized vesicular system. Fig. 2 displays the FTIR spectra of pure ciprofloxacin, phosphatidylcholine (PC), hyaluronic acid (HA), and the optimized formulation. The spectrum of pure ciprofloxacin revealed distinct vibrational bands consistent with its functional groups. A broad absorption band between 3450 and 3500 cm^−1^ was assigned to O—H stretching, while sharp peaks observed around 1700–1750 cm^−1^ corresponded to carbonyl (C=O) stretching vibrations. Additional signals between 1600 and 1650 cm^−1^ were attributed to aromatic ring vibrations of the quinolone nucleus, and a notable peak near 1050 cm^−1^ indicated C—F stretching, characteristic of fluoroquinolone structures (Arshad et al., 2021). Phosphatidylcholine exhibited absorption bands typical of phospholipid compounds. These included symmetric and asymmetric C—H stretching vibrations around 2850–2950 cm^−1^, ester carbonyl stretching at ∼1740 cm^−1^, and C–O–C ether vibrations near 1060 cm^−1^. These peaks confirmed the structural integrity of the phospholipid used in vesicle formation (Ahmed et al., 2024b). Hyaluronic acid displayed a broad band in the 3200–3600 cm^−1^ region due to O—H stretching, along with a sharp peak near 1730 cm^−1^, which was attributed to the stretching of the carboxylic acid C=O group. These features are indicative of the polysaccharide's hydrophilic nature and its potential to engage in hydrogen bonding (Servaty et al., 2001). In contrast, the FTIR spectrum of the optimized formula showed noticeable attenuation or complete disappearance of several ciprofloxacin-specific peaks (Elgendy et al., 2023; Ahmed et al., 2024b). This spectral modification indicates successful entrapment of the drug within the bilayers and suggests reduced free drug on the particle surface. The absence of new peaks further implies a lack of strong chemical interactions, supporting the physical encapsulation model (Hande et al., 2019; Sharma et al., 2023; Nemr et al., 2025).

#### 3.3.3 Assessment of mucoadhesive potential

The mucoadhesive capability of the optimized Hybrid Tri-Polymer Hyalurosomes was evaluated in the context of trans-tympanic application, aiming to ensure intimate and prolonged contact with the mucosal lining of the middle ear. This interaction is critical for enhancing drug retention at the tympanic site and facilitating effective trans-mucosal diffusion. Initial measurements of the mucin dispersion revealed a zeta potential (ZP) of −11.8 ± 3.14 mV, consistent with its natural anionic character. Upon incubation with the optimized hyalurosomal formulation, the ZP significantly shifted to −27.1 ± 1.27 mV. This substantial increase in negative charge reflects strong physicochemical interactions between the vesicles and the mucin layer. The heightened mucoadhesion is primarily attributed to the negatively charged surface of the vesicles, contributed by phosphatidylcholine and hyaluronic acid. Such interactions suggest a high binding affinity, enhancing formulation residence time within the ear canal and enabling sustained drug diffusion across the tympanic membrane (Saher et al., 2019; Albash et al., 2021; Said et al., 2021). These results highlight the formulation's promising potential as a trans-tympanic drug delivery system, offering localized and prolonged therapeutic effects with minimal systemic exposure.

#### 3.3.4 In vitro release profile and kinetic modelling

**Fig. 3:**
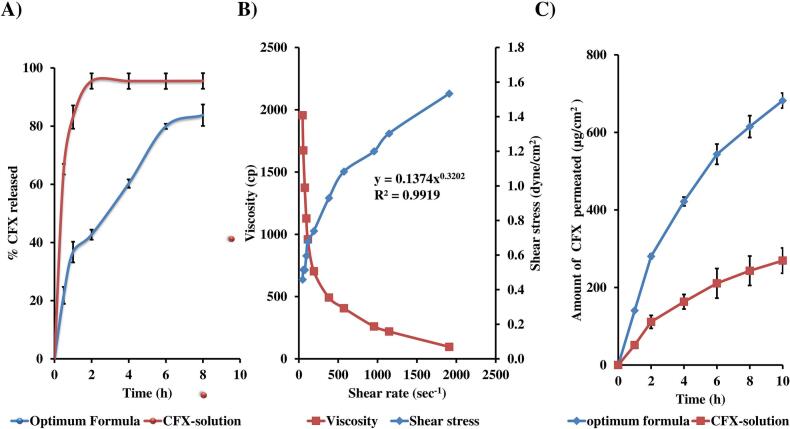
*In vitro* release (A), rheological characteristics (B) and *ex-vivo* permeation (C) profiles from optimum formula, demonstrating biphasic drug release, pseudo-plastic (shear-thinning) rheology, and enhanced trans-tympanic permeation.

An in vitro release study was conducted to compare the drug release behavior of the optimized hyalurosomal formulation with that of a plain ciprofloxacin solution. As illustrated in Fig. 3A, the free ciprofloxacin solution exhibited a rapid and nearly complete release (∼95 %) within the initial 2 h, confirming unimpeded drug diffusion through the dialysis membrane and minimal resistance from the release medium. In contrast, the optimized formulation demonstrated a biphasic release pattern characterized by an initial burst phase followed by a sustained release phase. During the first 2 h, approximately 43 % of the encapsulated ciprofloxacin was released, primarily due to the quick desorption of surface-bound or loosely associated drug molecules (Abd El-Alim et al., 2020). This was subsequently followed by a prolonged, slower release phase, reflecting the gradual diffusion of ciprofloxacin entrapped within the inner vesicular core and lipid bilayers. The sustained release profile is likely a result of the strong affinity of ciprofloxacin for the hydrophobic domains of the vesicular matrix, enhanced by the presence of phosphatidylcholine, HA and surfactants. These components collectively increase bilayer rigidity and reduce the formation of aqueous pores, thereby limiting drug diffusion (Abd-Elsalam and Ibrahim, 2021). Such a barrier effect contributes significantly to the formulation's ability to provide controlled release over an extended duration. Kinetic analysis confirmed that the drug release mechanism followed Higuchi's diffusion model, with a highest correlation coefficient, indicating that diffusion was the primary mode of release. This sustained release behavior offers potential therapeutic benefits by minimizing dosing frequency and ensuring prolonged drug availability at the site of application (Younes et al., 2024).

#### 3.3.5 Viscosity and flow characterization

The rheological assessment of the optimized hybrid tri-polymer hyalurosomal system revealed a pseudoplastic shear-thinning flow behavior, as depicted in Fig. 3B. As the applied shear rate increased, the viscosity of the formulation progressively decreased, a characteristic of non-Newtonian flow. Such rheological features offer significant functional benefits in the context of mucosal delivery. During application where mechanical stress is applied, the formulation becomes less viscous, facilitating smooth and uniform spreading across the mucosal surface. Upon cessation of shear (post-application), the viscosity increases, enabling the formulation to remain in place and resist premature leakage or clearance. This behavior is crucial for maintaining prolonged contact with the mucosal epithelium, enhancing both mucoadhesion and localized drug bioavailability (Ahmed et al., 2025f). Quantitative analysis using the power law model demonstrated a flow behavior index (n) of 0.3202, affirming the non-Newtonian, shear-thinning nature of the formulation. Moreover, the Carreau model yielded the best statistical fit to the experimental data, with an R^2^ value of 0.9919, further validating the pseudoplastic profile (Fahmy et al., 2018).

#### 3.3.6 Impact of storage

To evaluate the robustness of the developed hybrid hyalurosomal formulation, a comprehensive stability study was conducted under refrigerated conditions (5 ± 3 °C) over a period of three months. The formulation preserved its original physicochemical appearance, showing no signs of turbidity, precipitation, or particle aggregation, thus reflecting excellent physical stability. Key characteristics, including particle size (PS), entrapment efficiency (EE%) and zeta potential (ZP) remained statistically unaltered (*p >* 0.05), as outlined in Table 4, confirming the consistency of the formulation during storage. Moreover, the in vitro release profile of the stored formulation exhibited a strong resemblance to that of the freshly prepared batch, as evidenced by a similarity factor (f₂ = 76.88), which exceeded the acceptable threshold of 50 (Diaz et al., 2016; Ahmed et al., 2024a). This suggests that the formulation retained its sustained-release characteristics, reinforcing its potential for prolonged therapeutic action upon trans-tympanic application.

**Table 4:** Post-Storage Evaluation of the Optimized Formula.

Parameter	Fresh	Storage for 1 months at 4–8 °C	
		Value	Probability (*p*)⁎
EE %	90.28 ± 1.12	87.63 ± 0.78	0.111
PS	218.15 ± 2.33	213.90 ± 2.55	0.224
ZP	−40.40 ± 3.82	−39.30 ± 2.97	0.778

**Abbreviations:** EE %, percent entrapment efficiency; PS, particle size; ZP, zeta potential.

⁎ One-way ANOVA analysis to compare between the freshly prepared and the stored optimum formula.

The formulation's colloidal stability can be primarily ascribed to the highly negative zeta potential, which promotes electrostatic repulsion between vesicles and mitigates aggregation. This repulsive mechanism is further amplified by the nanoscale vesicle size, which increases the surface area and enhances charge exposure, contributing to prolonged dispersion uniformity. In addition to electrostatic stabilization, the inclusion of steric stabilizers and phospholipids imparts an additional layer of protection. These amphiphilic agents adsorb onto the vesicle surface, forming a hydrated shell that physically hinders vesicle–vesicle contact, thereby preventing fusion or coalescence. This synergistic combination of electrostatic and steric repulsion underpins the structural integrity of the nanosystem (Nemr et al., 2022; Ahmed et al., 2025b).

### 3.4 Microbiological efficacy assessment

#### 3.4.1 Evaluating the antibacterial efficacy through MIC and MBC determination

To evaluate the enhanced antimicrobial activity of the developed ciprofloxacin-loaded hyalurosomal formulation, minimum inhibitory concentration (MIC) and minimum bactericidal concentration (MBC) values were determined using the broth microdilution technique. The optimized formulation exhibited markedly enhanced antibacterial potency, evidenced by significantly lower MIC values against *Pseudomonas aeruginosa* PAO1 and *Staphylococcus aureus* USA300 (0.488 μg/mL and 0.976 μg/mL, respectively), when compared to the conventional ciprofloxacin solution (3.9 μg/mL and 7.8 μg/mL, respectively). Further bactericidal evaluation revealed that both the optimized nanosystem and the free drug exerted bactericidal effects after a 24-h incubation period at 37 °C. However, the hyalurosomal formulation outperformed the drug solution by achieving significantly reduced MBC values: 0.976 μg/mL for *Pseudomonas aeruginosa* PAO1 and 1.953 μg/mL for *Staphylococcus aureus* USA300, in contrast to 15.6 μg/mL for both strains when treated with the free drug.

These results strongly underscore the superior antibacterial efficacy of the optimized formula. The observed enhancement can be attributed to the improved permeation and sustained release provided by the vesicular carrier, enabling more effective interaction with bacterial cell membranes and maximized drug availability at the infection site. Such enhanced antimicrobial activity positions this nanosystem as a promising candidate for treating resistant or deep-seated infections, where localized delivery and retention are critical.

#### 3.4.2 Assessment of the biofilm inhibition

**Fig. 4:**
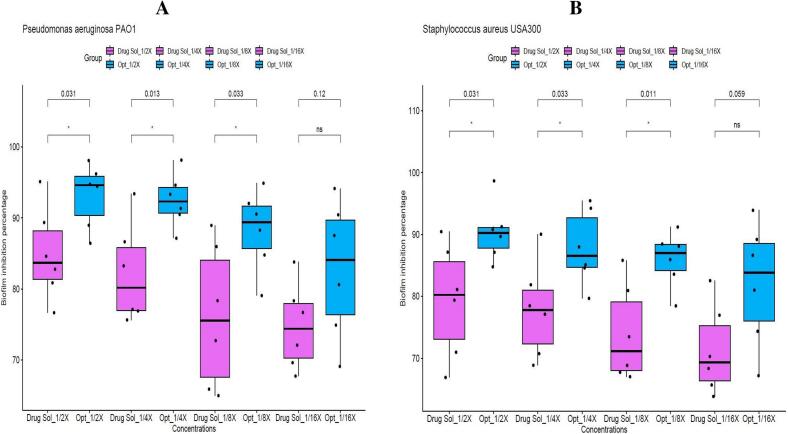
(A) Inhibitory effect of different concentrations (^1^/_16_, ^1^/_8_, ^1^/_4_, and ^1^/_2_ X, where X is the calculated MIC) of the optimized Hybrid Tri-Polymer Hyalurosomes (Opt) and ciprofloxacin solution (Drug Sol) on *Pseudomonas aeruginosa* PAO1 biofilm formation, demonstrating the improved microbiological activity of the optimum formula. Optimized formula concentrations are as follows:^1^/_16_X= 0.03 μg/mL, ^1^/_8_ X= 0.061 μg/mL, ^1^/_4_ X= 0.122 μg/mL, ^1^/_2_ X= 0.244 μg/mL. Drug solution concentrations are as follows**:**^1^/_16_X= 0.243 μg/mL, ^1^/_8_ X= 0.487 μg/mL, ^1^/_4_ X= 0.975 μg /mL, ^1^/_2_ X= 1.95 μg/mL. (B) Inhibitory effect of different concentrations (^1^/_16_, ^1^/_8_, ^1^/_4_, and ^1^/_2_ X, where X is the calculated MIC) of the optimized Hybrid Tri-Polymer Hyalurosomes (Opt) and ciprofloxacin solution (Drug Sol) on *Staphylococcus aureus* USA300 biofilm formation, demonstrating the improved microbiological activity of the optimum formula. Optimized formula concentrations are as follows:^1^/_16_X= 0.061 μg/mL, ^1^/_8_ X= 0.112 μg/mL, ^1^/_4_ X=0.244  μg /mL, ^1^/_2_ X=0.488 μg/mL. Drug solution concentrations are as follows:^1^/_16_X= 0.487 μg/mL, ^1^/_8_ X=0.975  μg/mL, ^1^/_4_ X= 1.95 μg/mL, ^1^/_2_ X= 3.9 μg/mL. The paired analysis was conducted within each concentration between optimized formula and the drug solution. Statistical significance was assessed by Student’s t-test, and all *p* values are shown.

To investigate the ability of the optimized ciprofloxacin-loaded hyalurosomal formulation to disrupt biofilm formation, a crystal violet staining assay was conducted. The biofilm inhibitory effects of the nanosystem and the plain ciprofloxacin solution were examined and compared at sub-inhibitory concentrations equivalent to ^1^/_16_, ^1^/_8_, ^1^/_4_, and ^1^/_2_ ×, where × represents the minimum inhibitory concentration previously determined. The results demonstrated that the hyalurosomal formulation exhibited significantly enhanced anti-biofilm activity against *Pseudomonas aeruginosa* PAO1 and *Staphylococcus aureus* USA300, at ^1^/_2_×, ^1^/_4_×, and ^1^/_8_× concentrations (Student's t-test, *p* < 0.05), as illustrated in Fig. 4A and B. This notable enhancement suggests that the vesicular formulation effectively hinders the establishment of bacterial biofilms likely due to improved penetration and prolonged interaction with the bacterial community. All statistical analyses were conducted using R software (version 4.1.2) and visualized via RStudio (Racine, 2012).

### 3.5 Integrated ex vivo and in vivo assessments in animals

#### 3.5.1 Ex vivo permeation

To evaluate the trans-tympanic permeation capabilities of the optimized Hybrid Tri-Polymer Hyalurosomes, an ex vivo diffusion study was conducted. As shown in Fig. 3C, the optimized Hyalurosomes significantly outperformed the ciprofloxacin (CFX) solution in terms of both the cumulative drug permeated and the permeation flux over a 10-h period. The optimized formulation achieved a Q_10_h of 682.23 ± 19.41 μg/cm^2^, markedly higher than the 269.28 ± 32.75 μg/cm^2^ recorded for the plain CFX solution. Similarly, the flux (J_max_) was elevated to 68.22 ± 1.94 μg/cm^2^/h, compared to only 26.93 ± 3.27 μg/cm^2^/h for the drug solution, corresponding to an enhancement ratio (ER) of 2.53 (*p* < 0.05).

This notable enhancement in trans-tympanic permeation can be attributed to several synergistic features of the Hybrid Hyalurosomes. First, their nano-scaled size increases surface area, enabling deeper membrane interaction and prolonged residence at the mucosal site (Mosallam et al., 2021a). Second, the negative zeta potential of the system promotes electrostatic repulsion between vesicles, ensuring colloidal stability and preventing aggregation, which supports sustained diffusion and interaction with the TM epithelium (Gilani et al., 2022). Moreover, the multi-functional excipients used in the formulation further contribute to its superior permeation profile. Phosphatidylcholine (PC), a key bilayer-forming agent, offers exceptional biocompatibility and promotes membrane fluidization, enhancing transcellular transport (Vater et al., 2019). The inclusion of Brij L4, a polyoxyethylene based non-ionic surfactant, reduces interfacial tension and potentially loosens tight junctions, thereby facilitating deeper epithelial penetration (Younes et al., 2024). Additionally, L121, a polymeric surfactant, improves bilayer deformability and vesicular elasticity, enabling the vesicles to traverse narrow intercellular spaces within the tympanic membrane structure (Ahmed et al., 2021). Importantly, the formulation also incorporates hyaluronic acid (HA), which contributes not only to mucoadhesiveness but also to prolonged localization at the trans-tympanic interface due to its ability to form hydrogen bonds (Fahmy et al., 2021). This prolonged contact further supports drug diffusion across the membrane. These results underscore the clinical promise of the optimized Hybrid Tri-Polymer Hyalurosomes as a non-invasive ototopical platform for improving ciprofloxacin delivery.

#### 3.5.2 Histological assessment of safety

**Fig. 5:**
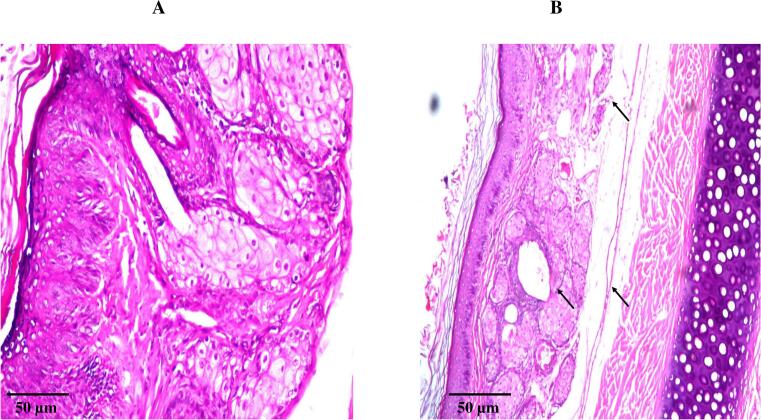
Histopathological sections of rabbits’ tympanic membrane after instillation of; (A) Normal saline solution (negative control) and (B) optimum formula, demonstrating normal structural integrity without signs of inflammation, edema, or cellular damage, confirming the biocompatibility and safety of the optimum formula.

To assess the biocompatibility and local tolerability of the Hybrid Tri-Polymer Hyalurosomes following trans-tympanic application, a histological examination of the rabbit tympanic membranes (TMs) was conducted. As shown in Fig. 5, the TM samples treated with normal saline (negative control) displayed intact and healthy epithelial layers, with no observable signs of cellular infiltration, edema, or structural alteration indicating a baseline of normal histological architecture. Strikingly, the TMs exposed to the optimized hyalurosomal formulation exhibited no histopathological abnormalities, inflammation, or signs of irritation. The epithelial integrity and connective tissue structures remained indistinguishable from the negative control group. These findings underscore the excellent local safety profile of the developed nanosystem when applied topically to the ear. This favorable outcome can be attributed to the careful selection and balanced composition of formulation excipients. Collectively, the absence of inflammatory or degenerative changes following application confirms the suitability of Hybrid Tri-Polymer Hyalurosomes for non-invasive AOM therapy, reinforcing their clinical promise as a safe and effective vehicle for middle ear drug targeting.

#### 3.5.3 CLSM-based in vivo trans-tympanic uptake

**Fig. 6:**
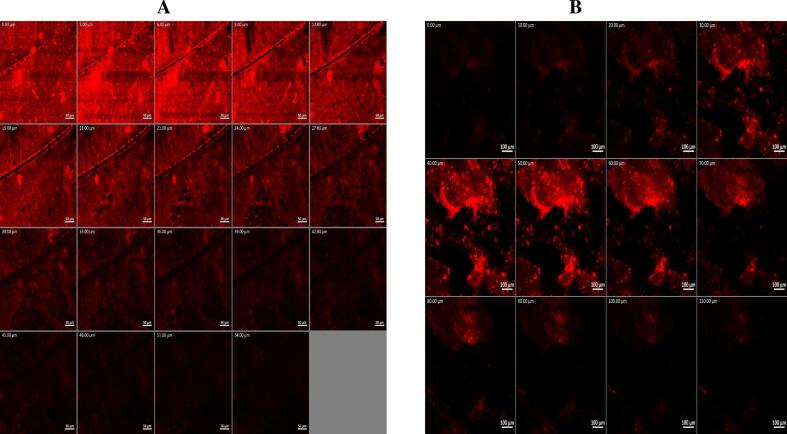
Confocal laser scanning micrographs of rabbits’ tympanic membrane after instillation of: (A) RhB aqueous solution and (B) RhB-loaded optimum formula, demonstrating deeper permeation of the optimum formula, indicative of enhanced permeation and delivery efficiency.

To further confirm the ex vivo permeation performance of the Hybrid Tri-Polymer Hyalurosomes, a confocal laser scanning microscopy (CLSM) study was performed to investigate in vivo penetration through the intact tympanic membrane (TM). As illustrated in Fig. 6, the RhB-labeled optimized formula exhibited significantly deeper tissue penetration compared to the free dye solution. The fluorescence signal from the hyalurosomal system extended to a depth of approximately 110 μm, while the control solution showed limited diffusion, reaching only 54 μm. This remarkable enhancement in penetration depth aligns well with the ex vivo findings and underscores the system's capability to traverse the TM barrier efficiently (Ahmed et al., 2025d). The observed enhancement is likely attributed to multiple synergistic factors: the nano-scale vesicle size facilitating closer interaction with the mucosal surface, the high deformability of the hybrid polymeric bilayer, and the presence of penetration-enhancing components such as phosphatidylcholine and surfactants (Ahmed et al., 2025f). Additionally, the mucoadhesive and hydrating effects of hyaluronic acid not only improved formulation retention at the site of administration but also facilitated closer contact and enhanced diffusion through the membrane layers (Fahmy et al., 2021). CLSM confirms that the Hybrid Hyalurosomes significantly improve trans-tympanic transport, offering promising implications for achieving therapeutic drug concentrations within the middle ear space.

## 4 Limitations and challenges

While our animal study design provided informative results, including more animals could have increased statistical power. However, we intentionally limited the number of animals to adhere to the 3R principles of animal research (reduce, reuse, and recycle). Furthermore, Long-term stability of the formulation was also not assessed and should be considered in future studies. Finally, this study was limited to assessing biofilm mass using crystal violet staining, which does not evaluate disruption of mature biofilms or bacterial viability. Future work should include viability-based assays such as CFU enumeration or metabolic activity (e.g., XTT or resazurin assays) to enhance clinical relevance.

## 5 Conclusions

The present study successfully introduced Novel Hybrid Tri-Polymer Hyalurosomes, a cutting-edge vesicular platform designed to overcome the limitations of conventional ototopical therapies and unlock the full potential of non-invasive trans-tympanic drug delivery. Through a systematic factorial optimization strategy, the developed system achieved exceptional entrapment efficiency, nanoscale particle size, robust zeta potential, and remarkable desirability, collectively ensuring its superior performance. The optimized formulation demonstrated spherical morphology, pseudoplastic rheology, and a bi-phasic sustained release profile, which together facilitate ease of administration, prolonged tympanic membrane contact, and controlled therapeutic availability. These attributes were further supported by enhanced mucoadhesive properties, excellent storage stability, and a 2.53-fold increase in drug permeation compared to conventional CFX solution. Crucially, the hybrid Hyalurosomes exhibited potent antibacterial and anti-biofilm activity, significantly reducing MIC and MBC values against both *Pseudomonas aeruginosa* and *Staphylococcus aureus*. Confocal laser scanning microscopy confirmed deeper membrane penetration, while histopathological evaluation demonstrated a favorable safety profile, establishing the system's suitability for ototopical use. Taken together, these findings highlight Novel Hybrid Tri-Polymer Hyalurosomes as a transformative platform capable of revolutionizing the management of acute otitis media and other middle ear infections. By combining multi-functional polymers, optimized nanostructure design, and comprehensive multi-scale validation, this innovative delivery system sets the stage for next-generation, patient-friendly, and clinically translatable therapeutics.

## CRediT authorship contribution statement

**Sadek Ahmed:** Resources, Investigation, Formal analysis, Conceptualization, Writing – original draft. **Heba Attia:** Resources, Investigation, Formal analysis, Conceptualization, Writing – original draft. **Osama Saher:** Resources, Formal analysis, Writing – review & editing.

## Funding

The current work has not received any form of funding from any source.

## Declaration of competing interest

The authors declare that they have no known competing financial interests or personal relationships that could have appeared to influence the work reported in this paper.

## Data Availability

The datasets generated during and/or analyzed during the current study are available from the corresponding author on reasonable request.
